# Integrative Biology of Diabetic Retinal Disease: Lessons from Diabetic Kidney Disease

**DOI:** 10.3390/jcm10061254

**Published:** 2021-03-18

**Authors:** Warren W. Pan, Thomas W. Gardner, Jennifer L. Harder

**Affiliations:** 1 Department of Ophthalmology and Visual Sciences, University of Michigan Medical School, Ann Arbor, MI 48105, USA; warrenp@med.umich.edu (W.W.P.); tomwgard@med.umich.edu (T.W.G.); 2 Department of Internal Medicine (Metabolism, Endocrinology and Diabetes), University of Michigan Medical School, Ann Arbor, MI 48109, USA; 3 Department of Molecular and Integrative Physiology, University of Michigan Medical School, Ann Arbor, MI 48109, USA; 4 Department of Internal Medicine (Nephrology), University of Michigan Medical School, Ann Arbor, MI 48109, USA

**Keywords:** diabetic retinal disease, diabetic kidney disease, systems biology, diabetic retinopathy, microangiopathy, neurovascular unit, treatment of diabetic retinopathy, clinical aspects of diabetic retinopathy, research in diabetic retinopathy

## Abstract

Diabetic retinal disease (DRD) remains the most common cause of vision loss in adults of working age. Progress on the development of new therapies for DRD has been limited by the complexity of the human eye, which constrains the utility of traditional research techniques, including animal and tissue culture models—a problem shared by those in the field of kidney disease research. By contrast, significant progress in the study of diabetic kidney disease (DKD) has resulted from the successful employment of systems biology approaches. Systems biology is widely used to comprehensively understand complex human diseases through the unbiased integration of genetic, environmental, and phenotypic aspects of the disease with the functional and structural manifestations of the disease. The application of a systems biology approach to DRD may help to clarify the molecular basis of the disease and its progression. Acquiring this type of information might enable the development of personalized treatment approaches, with the goal of discovering new therapies targeted to an individual’s specific DRD pathophysiology and phenotype. Furthermore, recent efforts have revealed shared and distinct pathways and molecular targets of DRD and DKD, highlighting the complex pathophysiology of these diseases and raising the possibility of therapeutics beneficial to both organs. The objective of this review is to survey the current understanding of DRD pathophysiology and to demonstrate the investigative approaches currently applied to DKD that could promote a more thorough understanding of the structure, function, and progression of DRD.

## 1. Introduction

**Figure 1:**
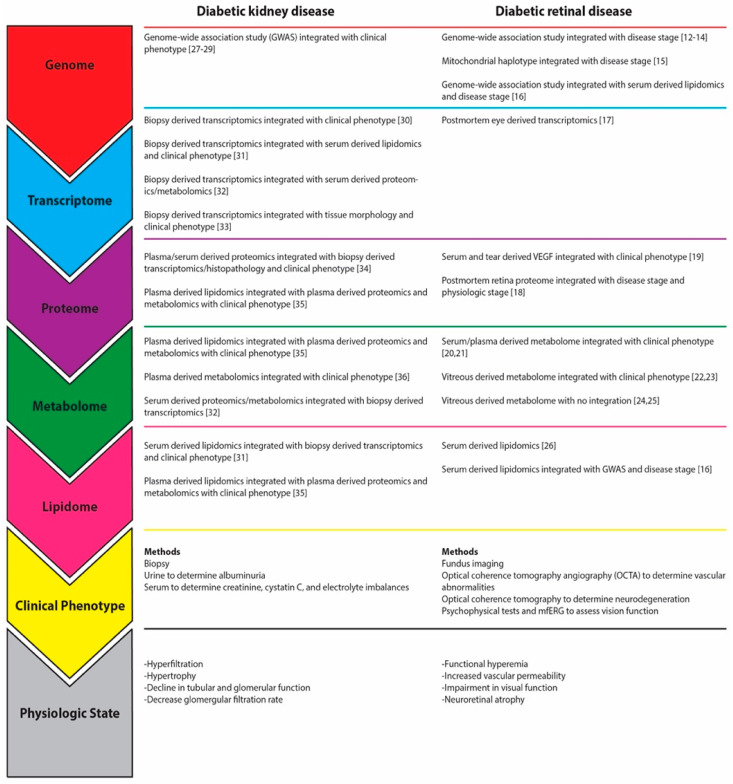
Systems Biology of diabetic retinal disease (DRD) and diabetic kidney disease (DKD): Integration of Multi-Scalar Data. Individual studies examining either diabetic kidney disease (**left** column) or diabetic retinal disease (right column) are listed based on the different types of data labeled vertically on the left, demonstrating downward multi-scalar integration of data [12,13,14,15,16,17,18,19,20,21,22,23,24,25,26,27,28,29,30,31,32,33,34,35,36]. Additionally, the methods and physiologic manifestations of the disease are listed as clinical phenotype and physiologic state, respectively.

Diabetic Retinal Disease (DRD) is a complication of diabetes responsible for significant morbidity and decreased productivity and quality of life [1,2]. The growing burden of DRD has accompanied the epidemic growth in prevalence of diabetes, which has quadrupled over the last four decades from 108 million in 1980 to over 425 million worldwide today [3,4]. The International Diabetes Federation projects these numbers will rise to 578 million by 2030 and 700 million by 2045 [5]. Despite the increase in DRD disease burden, current pharmacologic approaches are limited to laser therapy and intravitreally injected anti-vascular endothelial growth factor (VEGF) agents due largely to our limited understanding of DRD pathophysiology [6,7,8,9]. A comprehensive understanding of the molecular underpinnings of DRD, however, is limited by the use of traditional research techniques, including cell culture and rodent models that do not recapitulate human DRD [10]. Thus, there is a significant unmet need for innovative research approaches to reveal insights about DRD in humans that are not captured by model systems to inspire the next wave of DRD therapies. Recent Diabetic Kidney Disease (DKD) studies using the systems biology approach, which integrates different types of data, including genomics, epigenomics, transcriptomics, proteomics, metabolomics, and phenomics (the systemic study of phenotypes), have deepened our understanding of DKD pathogenesis (Figure 1). The discovery of unanticipated insights about disease pathomechanisms is enhanced by the use of agnostic bioinformatics analysis methods in systems biology. Systems biology is rapidly becoming a valuable approach in the field of DRD research as well [11,12,13,14,15,16,17,18,19,20,21,22,23,24,25,26]. Thus, our purpose is to detail the pathophysiologic understanding of DRD and highlight the potential role of systems biology to innovate this approach clinically and scientifically in the study and management of DRD.

## 2. DRD: More Than a Vasculopathy

**Figure 2:**
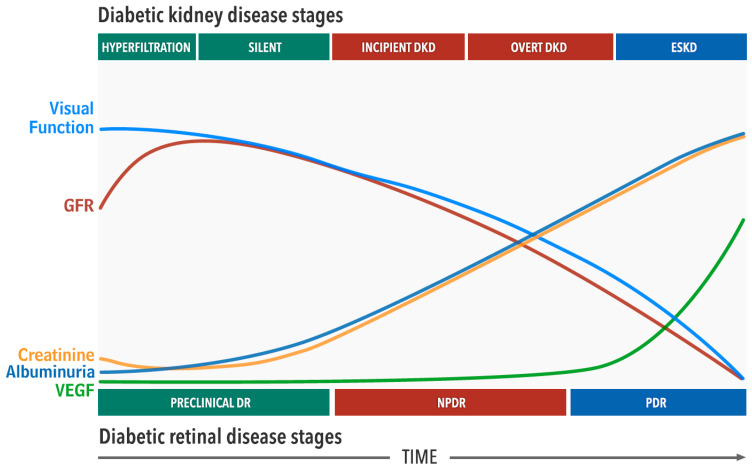
The parallel processes of diabetic kidney and retinal disease development and progression demonstrate an opportunity to identify early pathomechanisms of DRD. This figure illustrates a key aspect shared by DRD and DKD: As time progresses from onset of diabetes and adaptive measures have failed, progressive disease ensues (from left to right over time). DKD researchers have recently identified molecular pathways active in early DKD (green bars, top left of chart) that are associated with increased risk for disease progression before classic markers such as albuminuria, serum creatinine, or GFR are affected. By striving to characterize better the molecular pathways active in the analogous preclinical period of DRD (green bar, bottom left of chart) that are associated with progression before VEGF has a central role and visual function is impaired, targeted therapies halting or reversing early disease may become possible. The stages of DKD (top row) progress from the hyperfiltration stage to the silent, incipient, overt DKD stages and ultimately to end-stage kidney disease (ESKD). The stages of DRD (bottom row) progress from preclinical diabetic retinopathy to non-proliferative DR, to proliferative DR. Biomarkers including albuminuria (navy line), creatinine (yellow line), and VEGF (green line) help diagnose the stage of the disease and determine the estimated kidney function by GFR (red line). Similarly, a hypothetical visual function (blue line) could potentially be estimated. GFR: Glomerular filtration rate, VEGF: Vascular endothelial growth factor, DR: Diabetic retinopathy, NPDR: Non-proliferative diabetic retinopathy, PDR: Proliferative diabetic retinopathy.

The current standard for DRD diagnosis relies on the decades-old Early Treatment Diabetic Retinopathy Study (ETDRS) grading scale based exclusively on vascular abnormalities [37,38,39,40]. Thus, most DRD evaluations rely on seven-field fundus photographs [41,42], which reconstructs the inner retina surface by reflecting white light with 30-degree fields of view from the posterior pole to determine the level of retinopathy present, such as no retinopathy, non-proliferative, or proliferative diabetic retinopathy, and the presence or absence of diabetic macular edema [43] (Figure 2). Consistent with this focus, laser therapy, VEGF inhibitors like bevacizumab (off-label), ranibizumab, aflibercept, and brolucizumab, and corticosteroids such as dexamethasone intravitreal implant comprise the current therapies for DRD [6,7,8]. However, the application of methods to better define the DRD clinical phenotype (Figure 1) has called this vascular-centric view into question [44,45].

Psychophysical tests, a series of well-validated assessments, have found a subset of patients to have deficits in peripheral vision, night vision, color-hue discrimination, and contrast discrimination during the preclinical DR stage, before the development of vascular abnormalities [46,47,48,49]. These functional deficits were further corroborated by abnormal measurements revealed by multifocal electroretinography (mfERG), an electrophysiologic test of cone photoreceptor pathways in the retina, in patients with preclinical DRD [50,51,52,53]. The observed functional deficits were also directly linked to neuronal layer thinning observed by optical coherence tomography (OCT) [54,55], which captures and reconstructs a cross-section of the different retinal layers on a cellular resolution [56]. Together, these clinical phenotype modalities suggest that at least a subset of persons with diabetes experience visual function loss primarily from neurodegeneration [57,58,59], perhaps independent of the vascular structural abnormalities observed in NPDR (Non-proliferative diabetic retinopathy) and PDR (Proliferative diabetic retinopathy) [60,61]. The relationship between these phenotypes warrants further investigation [62,63]. To this end, visual function testing such as contrast sensitivity and visual fields are secondary endpoints in large clinical trials (NCT04661358 and NCT042655261), and a new DRD grading scale is being developed that will include visual function testing as an integral component [64].

## 3. DKD: Another Frequent Comorbidity in Diabetes

Like DRD, DKD has historically been viewed as a vasculopathy responsible for significant morbidity and mortality in diabetes. Indeed, DKD is consistently the most common cause of end-stage kidney disease (ESKD) in the United States [65,66]. A recent estimate concluded that chronic kidney disease affects about 9% of diabetics aged 22–64 and about 30% of diabetics aged 65 and above [2]. Similar to DRD, DKD progression is classically described as a series of characteristic findings; hyperfiltration (an adaptive mechanism to maintain glomerular filtration rate (GFR)) results in kidney hypertrophy, followed by the development of albuminuria and loss of GFR (Figure 2). The clinical assignment of DKD stage relies on serum and urine tests to monitor creatinine, cystatin C, electrolyte imbalances, and albuminuria, and sometimes accompanied by kidney biopsy histology (Figure 1).

However, this classic DKD description does not capture the wider clinical variability of this disease. While some studies have estimated microalbuminuria to have an 80% predictive value for DKD progression [67,68], newer estimates suggest that DKD progression occurs in only 30–45% of patients with microalbuminuria [69]. In addition, accumulating evidence indicates that progression of kidney disease can also occur in individuals with type 1 or type 2 diabetes without albuminuria [70]. Lack of albuminuria in DKD is analogous to preclinical DRD causing vision loss via neurodegeneration, thus implicating multiple potential pathomechanisms involved in the development of both DKD and DRD.

This variation in DKD and DRD presentations and the existence of multiple phenotypes complicates our ability to understand, predict, and treat this disease, necessitating novel research approaches to determine the basis for this complexity. Such efforts are paramount in order to advance the current DKD treatment paradigm, which largely remains unchanged since the discovery of angiotensin receptor inhibition in the 1990s and consists of glycemic control, blood pressure management, fluid balance, and angiotensin-converting enzyme (ACE) inhibition [71,72,73,74,75,76,77]. Indeed, our diagnostic and therapeutic understanding of DKD is now advancing with the incorporation of systems biology approaches [27,28,29,30,31,32,33,34,35,36,78].

## 4. Systems Biology Yields Insights into Pathomechanisms of DKD

In contrast to reductionist investigations that focus on single or a few molecules, the power of systems biology lies in its unbiased and agnostic multi-scalar integration of data generated from genomics, transcriptomics, proteomics, metabolomics, and lipidomics studies, along with tissue morphometry and clinical data and biomarkers to interrogate the true physiologic state of individuals in health and disease [11] (Figure 1). Systems biology has been integral in the targeted diagnostics of population subsets, especially helpful for DKD research given the variation of phenotypes. In a study of Pima Indians with diabetes and normal kidney function without albuminuria who were at high risk of developing progressive DKD, kidney tissue demonstrated early transcriptional pathway alterations that correlated with structural changes—findings that predicted GFR decline or development of albuminuria over the subsequent decade [33]. Agnostic pathway analysis also revealed enrichment of transcripts in pathways associated with mitochondrial dysfunction, inflammation, and tubular metabolic dysfunction, implicating these pathways in early DKD pathogenesis.

Systems biology studies have identified trackable biomarkers that correlate closely with structural and functional abnormalities early in DKD, including urine haptoglobin [79], urinary collagen fragments [80], epidermal growth factor (EGF) [30], and urine monocyte chemoattractant protein-1 (MCP-1) [81]. These urinary biomarkers can serve as readouts for the transcriptional networks involved in DKD pathogenesis and may be useful to create a new framework to assess early DKD, especially during the timeframe when current methods of defining clinical phenotype such as albuminuria or decrease in GFR are not detectable [69].

In addition to these advances to diagnostics [35], systems biology has also revealed insights on DKD pathophysiology that have paved the way for targeted therapeutics. Indeed, identification of inflammatory processes in early DKD with the involvement of the Janus Kinase/Signal Transducer and Activator of Transcription Pathway (JAK/STAT) pathway [82,83] led directly to successful clinical trials with baricitinib, a potent inhibitor of this pathway, repurposed from its FDA approved role as a therapeutic for rheumatoid arthritis [84]. The potent suppression of albuminuria with baricitinib treatment [77] suggests that its efficacy may be tied to the population of individuals who experienced albuminuria with DKD and/or DRD.

Together, the recent progress in DKD research highlights a new framework by which advances in personalized diagnostics and novel therapeutics can intervene at a time point early enough when disease progression is reversible. Figure 1 lists the materials used for systems biology analysis in DKD, consisting of biopsies that define kidney structure and easily accessible urine and serum. The overlap of material sources of clinical phenotyping and systems biology lends itself to the multi-scalar integration of biopsy-derived transcriptomics with serum-derived proteomics and metabolomics (Figure 1), allowing researchers to evaluate the overall efficacy of medications like dapagliflozin [32].

This approach is in contrast to DRD clinical phenotyping modalities, for which obtaining biosamples for systems biology analyses is not part of clinical care (Figure 1). One potential method is vitreous humor sampling of patients, which could provide information about vitreous proteins and lipids, as well as the transcriptional state of cells that have dislodged from the retinal surface. Indeed, vitreous samples are sometimes available for biochemical analysis when patients are undergoing vitrectomy surgery for clinical disease [85,86]. In combination with retinal transcriptional analysis in diabetic rodents [17] and proteomic analysis of post-mortem human retinas [18], a systems biology application to DRD research appears imminently feasible. Combining DRD datasets with existing datasets from other diabetes-centric studies, such as serum lipidomic studies and DKD-specific studies, provides an opportunity to broaden and deepen understanding of common and tissue-specific alterations induced by diabetes.

## 5. Shared Pathophysiology of DRD and DKD: An Avenue for Further Investigation

“Diabetic renal-retinal syndrome” describes the clinical phenomena of: (1) DRD vascular abnormalities as predictors of impaired renal function [87,88]; and (2) microalbuminuria as an accurate biomarker of DRD progression [89]. DKD and DRD share pathophysiologic changes between their analogous structures, such as loss of endothelial glycocalyx, a hallmark of early DKD progression, which has similarly been observed in DRD [90,91,92]. Likewise, basement membrane (BM) thickening of blood vessels is an early finding [93,94] thought secondary to increased levels of transforming growth factor-beta (TGF-β) in the kidneys [94,95,96] and retina [97]. These shared findings suggest a common pathomechanism of DKD and DRD. However, there are also clear differences between these pathomechanisms, since DRD and DKD do not always coincide [98]. The common and unique aspects of DKD and DRD emphasize the cross-applicability of DRD diagnostics applied to DKD categorization and vice versa.

**Figure 3:**
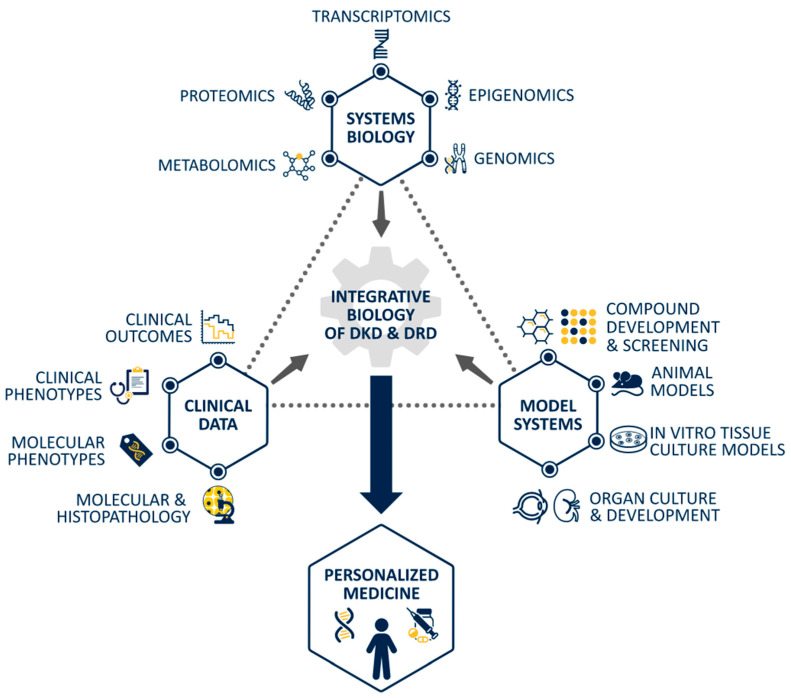
Integrative biology towards personalized medicine. Three main domains of research are depicted and include clinical data, model systems, and systems biology. Within each domain, multiple nodes of scientific research are detailed. For example, the systems biology domain includes genomics, epigenomics, transcriptomics, proteomics, and metabolomics research. The combination of these three domains contributes to integrative knowledge of pathomechanisms of disease, which when applied to both DKD and DRD culminates in personalized medicine.

Indeed, an important step forward in accurately assessing DKD and DRD lies in determining the spectrum of disease phenotypes [57,58,59,70] via patient/population-specific systems biology analyses across organs towards personalized diagnostic and therapeutic delivery (Figure 3). In this way, systems biology findings would be able to not just vertically integrate -omics data but also horizontally connect DRD findings with DKD discoveries (Figure 3. With this approach, the potential to precisely determine individual diabetes pathophysiology may be realized using a combination of kidney biopsies, retinal imaging modalities, and biomarkers like albuminuria [89], creatinine, and vascular endothelial growth factor (VEGF) [99] (Figure 2). Thus, the human-centric findings from systems biology may be used to redefine human disease and create a novel framework to develop new hypotheses that can be tested with different clinical and animal models (Figure 3).

The ultimate manifestation of intimate collaboration is the development of novel therapeutics for the complications of diabetes. Historically, the Diabetes Control and Complications Trial/Epidemiology of Diabetes Interventions and Complications (DCCT/EDIC), UK Prospective Diabetes Study (UKPDS), and Action to Control Cardiovascular Risk in Diabetes (ACCORD) trials established the importance of glycemic, blood pressure, and dyslipidemia control in DRD therapy [100,101,102,103,104]. Many of these findings, along with the importance of the renin-angiotensin system in DRD and DKD [104,105,106], have improved the standard of care for both complications. Interestingly, the DCCT emphasized the comparable magnitude of effect on the eyes, kidneys, and nerves from the same treatment [100], supporting the viability of organ-agnostic diagnosis and treatment.

However, systems biology-driven insights offer an alternative approach to therapeutics development. One example is the aforementioned JAK-STAT pathway affected in DKD. As the JAK-STAT pathway is also affected in DRD [107], it is possible that baricitinib [77], or other ocular therapeutics that act on this JAK-STAT pathway may become viable treatment options for DRD. If useful in DRD treatment, baricitinib would continue the history of discovering effective DRD therapies from clinical trials focused outside of the eye.

Regardless of whether baricitinib-mediated suppression of albuminuria [77] translates to novel DRD medications, this possibility emphasizes the need for the further identification and development of appropriate clinical endpoints to be used in DRD studies. The importance of additional endpoints is highlighted by the cautionary note that prolonged VEGF inhibition may accelerate retinal degeneration [108]. It is unclear whether the accelerated loss of peripheral visual field sensitivity after 5 years of ranibizumab [109] is due to DRD progression and/or due to the therapy. While the need for additional endpoints has been reaffirmed [110], and efforts to include mfERG and psychophysical tests are underway [111,112] (NCT04265261), the selection of additional endpoints beyond the eye may prove to be particularly important, as demonstrated by DCCT/EDIC, UKPDS, and ACCORD [100,101,102,103,104].

As these systems biology studies integrate the various levels of data (Figure 1), it becomes increasingly important to recognize the implications of novel diagnostics and therapeutics on the management of diabetes across organ systems and to have the endpoints necessary to monitor progress. Indeed, JDRF supports research for the express purpose of building on common avenues of investigation between DRD and DKD to make shared discoveries possible [113]. The inclusion of research, diagnostics, and therapeutics across organ systems will allow clinicians to identify and treat diabetic complications based on individual pathophysiology.

## 6. Conclusions: Where Do We Go from Here?

The fields of DRD and DKD have progressed dramatically since their categorizations as complications of diabetes. With the use of systems biology, new signaling pathways, biomarkers, and therapeutic targets have been identified for both diseases. However, there continues to exist a need to integrate these findings into the clinical evaluation and treatment of DRD and DKD. By allowing these two diseases to walk in tandem with multi-scalar investigation and assessment scientifically and medically, new strategies effective in the care of diabetic patients may be revealed.

Progress in DRD research and new therapies has been exceedingly slow with the reliance on classic reductionist scientific methods. Given the recent boon in development of systems biology techniques applied to other complex diseases such as DKD, DRD researchers can now take advantage of these transformative technologies. By employing agnostic analytic methods to big datasets, DRD research has an unparalleled and unbridled opportunity to reveal novel pathomechanisms involved in human DRD. Armed with more specific knowledge of human pathomechanisms of DRD, researchers will then be able to reapproach model systems to determine which model most closely recapitulates human biology of the research question to be asked (Figure 3). Furthermore, systems biology analysis of classic and novel model systems such as human stem cell-derived retinal organoids can help determine which model systems could be utilized for further hypothesis-driven mechanistic studies to validate these human-derived data. In DKD research, for example, interrogation of human kidneys and several mouse models of DKD revealed transcriptional networks unique to each mouse model, which was shared in human tissue, allowing researchers to choose a model more pertinent to their interest, as well as helping to explain earlier varied results among models [114].

Towards this goal, recent progress in human stem-cell-derived retinal organoids [115] provides a mechanism to study patient-specific aspects of disease and allows personalized screening of potential therapeutics ex vivo. By recapitulating an individual’s diseased tissue with the individual’s specific genetic background ex vivo, ophthalmologic researchers are advancing the National Institutes of Health’s (NIH) mandate to accelerate the development and application of precision medicine. Indeed, the NIH National Center for Advancing Translational Sciences’ (NCAT) Tissue Chip initiatives [116] are actively funding the development of such approaches. The Tissue Chip Development, Tissue Chips for Modeling Diabetes, and Clinical Trials on a Chip projects are aimed at the development and implementation of microphysiologic systems recapitulating tissue architecture and function. Integration of tissue chip technologies within clinic trials will soon have real-life patient impact. Clearly, the transformation of the field of DRD research is underway.

## References

[1] Yau J.W.Y., Rogers S.L., Kawasaki R., Lamoureux E.L., Kowalski J.W., Bek T., Chen S.J., Dekker J.M., Fletcher A., Grauslund J. (2012). Global prevalence and major risk factors of diabetic retinopathy. Diabetes Care.

[2] Saran R., Robinson B., Abbott K.C., Bragg-Gresham J., Chen X., Gipson D., Gu H., Hirth R.A., Hutton D., Jin Y. (2019). US Renal Data System 2019 Annual Data Report: Epidemiology of Kidney Disease in the United States.

[3] World Health Organization Diabetes. https://www.who.int/news-room/fact-sheets/detail/diabetes.

[4] Internation Diabetes Federation (2019). IDF Diabetes Atlas.

[5] Saeedi P., Petersohn I., Salpea P., Malanda B., Karuranga S., Unwin N., Colagiuri S., Guariguata L., Motala A.A., Ogurtsova K. (2019). Global and regional diabetes prevalence estimates for 2019 and projections for 2030 and 2045: Results from the International Diabetes Federation Diabetes Atlas, 9th edition. Diabetes Res. Clin. Pract..

[6] Googe J., Brucker A.J., Bressler N.M., Qin H., Aiello L.P., Antoszyk A., Beck R.W., Bressler S.B., Ferris F.L., Diabetic Retinopathy Clinical Research Network (2011). Randomized trial evaluating short-term effects of intravitreal ranibizumab or triamcinolone acetonide on macular edema after focal/grid laser for diabetic macular edema in eyes also receiving panretinal photocoagulation. Retina.

[7] Nguyen Q.D., Brown D.M., Marcus D.M., Boyer D.S., Patel S., Feiner L., Gibson A., Sy J., Rundle A.C., Hopkins J.J. (2012). Ranibizumab for diabetic macular edema: Results from 2 phase iii randomized trials: RISE and RIDE. Ophthalmology.

[8] Mitchell P., Bandello F., Schmidt-Erfurth U., Lang G.E., Massin P., Schlingemann R.O., Sutter F., Simader C., Burian G., Gerstner O. (2011). The RESTORE study: Ranibizumab monotherapy or combined with laser versus laser monotherapy for diabetic macular edema. Ophthalmology.

[9] Gillies M.C., Lim L.L., Campain A., Quin G.J., Salem W., Li J., Goodwin S., Aroney C., McAllister I.L., Fraser-Bell S. (2014). A randomized clinical trial of intravitreal bevacizumab versus intravitreal dexamethasone for diabetic macular edema: The BEVORDEX study. Ophthalmology.

[10] Olivares A.M., Althoff K., Chen G.F., Wu S., Morrisson M.A., DeAngelis M.M., Haider N. (2017). Animal Models of Diabetic Retinopathy. Curr. Diab. Rep..

[11] Harder J.L., Hodgin J.B., Kretzler M. (2015). Integrative Biology of Diabetic Kidney Disease. Kidney Dis..

[12] Liu E., Kaidonis G., McComish B.J., Gillies M.C., Abhary S., Essex R.W., Chang J.H., Pal B., Daniell M., Lake S. (2019). MicroRNA-related genetic variants are associated with diabetic retinopathy in type 1 diabetes mellitus. Investig. Ophthalmol. Vis. Sci..

[13] Pollack S., Igo R.P., Jensen R.A., Christiansen M., Li X., Cheng C.Y., Ng M.C.Y., Smith A.V., Rossin E.J., Segrè A.V. (2019). Multiethnic genome-wide association study of diabetic retinopathy using liability threshold modeling of duration of diabetes and glycemic control. Diabetes.

[14] Vuori N., Sandholm N., Kumar A., Hietala K., Syreeni A., Forsblom C., Juuti-Uusitalo K., Skottman H., Imamura M., Maeda S. (2019). CaCNB2 is a novel susceptibility gene for diabetic retinopathy in type 1 diabetes. Diabetes.

[15] Mitchell S.L., Neininger A.C., Bruce C.N., Chocron I.M., Bregman J.A., Estopinal C.B., Muhammad A., Umfress A.C., Jarrell K.L., Warden C. (2017). Mitochondrial haplogroups modify the effect of diabetes duration and HbA1c on proliferative diabetic retinopathy risk in patients with type 2 diabetes. Investig. Ophthalmol. Vis. Sci..

[16] Sobrin L., Chong Y.H., Fan Q., Gan A., Stanwyck L.K., Kaidonis G., Craig J.E., Kim J., Liao W.L., Huang Y.C. (2017). Genetically determined plasma lipid levels and risk of diabetic retinopathy: A mendelian randomization study. Diabetes.

[17] Platania C.B.M., Leggio G.M.G.M., Drago F., Salomone S., Bucolo C. (2018). Computational systems biology approach to identify novel pharmacological targets for diabetic retinopathy. Biochem. Pharmacol..

[18] Sundstrom J.M., Hernández C., Weber S.R., Zhao Y., Dunklebarger M., Tiberti N., Laremore T., Simó-Servat O., Garcia-Ramirez M., Barber A.J. (2018). Proteomic analysis of early diabetic retinopathy reveals mediators of neurodegenerative brain diseases. Investig. Ophthalmol. Vis. Sci..

[19] Ang W.J., Zunaina E., Norfadzillah A.J., Raja-Norliza R.O., Julieana M., Ab-Hamid S.A., Mahaneem M. (2019). Evaluation of vascular endothelial growth factor levels in tears and serum among diabetic patients. PLoS ONE.

[20] Li X., Luo X., Lu X., Duan J., Xu G. (2011). Metabolomics study of diabetic retinopathy using gas chromatography-mass spectrometry: A comparison of stages and subtypes diagnosed by Western and Chinese medicine. Mol. Biosyst..

[21] Chen L., Cheng C.Y., Choi H., Ikram M.K., Sabanayagam C., Tan G.S.W., Tian D., Zhang L., Venkatesan G., Tai E.S. (2016). Plasma metabonomic profiling of diabetic retinopathy. Diabetes.

[22] Tomita Y., Cagnone G., Fu Z., Cakir B., Kotoda Y., Asakage M., Wakabayashi Y., Hellström A., Joyal J.-S., Talukdar S. (2020). Vitreous metabolomics profiling of proliferative diabetic retinopathy. Diabetologia.

[23] Haines N.R., Manoharan N., Olson J.L., D’Alessandro A., Reisz J.A. (2018). Metabolomics Analysis of Human Vitreous in Diabetic Retinopathy and Rhegmatogenous Retinal Detachment. J. Proteome Res..

[24] Barba I., Garcia-Ramírez M., Hernández C., Alonso M.A., Masmique L., García-Dorado D., Simó R. (2010). Metabolic fingerprints of proliferative diabetic retinopathy: An 1H-NMR-based metabonomic approach using vitreous humor. Investig. Ophthalmol. Vis. Sci..

[25] Paris L.P., Johnson C.H., Aguilar E., Usui Y., Cho K., Hoang L.T., Feitelberg D., Benton H.P., Westenskow P.D., Kurihara T. (2016). Global metabolomics reveals metabolic dysregulation in ischemic retinopathy. Metabolomics.

[26] Xuan Q., Zheng F., Yu D., Ouyang Y., Zhao X., Hu C., Xu G. (2020). Rapid lipidomic profiling based on ultra-high performance liquid chromatography–mass spectrometry and its application in diabetic retinopathy. Anal. Bioanal. Chem..

[27] Sandholm N., Forsblom C., Mäkinen V.P., McKnight A.J., Österholm A.M., He B., Harjutsalo V., Lithovius R., Gordin D., Parkkonen M. (2014). Genome-wide association study of urinary albumin excretion rate in patients with type 1 diabetes. Diabetologia.

[28] Salem R.M., Todd J.N., Sandholm N., Cole J.B., Chen W.M., Andrews D., Pezzolesi M.G., Mc P.M.K., Hiraki L.T., Qiu C. (2019). Genome-Wide association study of diabetic kidney disease highlights biology involved in glomerular basement membrane collagen. J. Am. Soc. Nephrol..

[29] Sandholm N., Van Zuydam N., Ahlqvist E., Juliusdottir T., Deshmukh H.A., Rayner N.W., Di Camillo B., Forsblom C., Fadista J., Ziemek D. (2017). The genetic landscape of renal complications in type 1 diabetes. J. Am. Soc. Nephrol..

[30] Ju W., Nair V., Smith S., Zhu L., Shedden K., Song P.X.K., Mariani L.H., Eichinger F.H., Berthier C.C., Randolph A. (2015). Tissue transcriptome-driven identification of epidermal growth factor as a chronic kidney disease biomarker. Sci. Transl. Med..

[31] Afshinnia F., Nair V., Lin J., Rajendiran T.M., Soni T., Byun J., Sharma K., Fort P.E., Gardner T.W., Looker H.C. (2019). Increased lipogenesis and impaired B-oxidation predict type 2 diabetic kidney disease progression in American Indians. JCI Insight.

[32] Mulder S., Hammarstedt A., Nagaraj S.B., Nair V., Ju W., Hedberg J., Greasley P.J., Eriksson J.W., Oscarsson J., Heerspink H.J.L. (2020). A metabolomics-based molecular pathway analysis of how the sodium-glucose co-transporter-2 inhibitor dapagliflozin may slow kidney function decline in patients with diabetes. Diabetes Obes. Metab..

[33] Nair V., Komorowsky C.V., Weil E.J., Yee B., Hodgin J., Harder J.L., Godfrey B., Ju W., Boustany-Kari C.M., Schwarz M. (2018). A molecular morphometric approach to diabetic kidney disease can link structure to function and outcome. Kidney Int..

[34] Niewczas M.A., Pavkov M.E., Skupien J., Smiles A., Md Dom Z.I., Wilson J.M., Park J., Nair V., Schlafly A., Saulnier P.J. (2019). A signature of circulating inflammatory proteins and development of end-stage renal disease in diabetes. Nat. Med..

[35] Kammer M., Heinzel A., Willency J.A., Duffin K.L., Mayer G., Simons K., Gerl M.J., Klose C., Heinze G., Reindl-Schwaighofer R. (2019). Integrative analysis of prognostic biomarkers derived from multiomics panels helps discrimination of chronic kidney disease trajectories in people with type 2 diabetes. Kidney Int..

[36] Tofte N., Vogelzangs N., Mook-Kanamori D., Brahimaj A., Nano J., Ahmadizar F., van Dijk K.W., Frimodt-Møller M., Arts I., Beulens J.W.J. (2020). Plasma Metabolomics Identifies Markers of Impaired Renal Function: A Meta-analysis of 3089 Persons with Type 2 Diabetes. J. Clin. Endocrinol. Metab..

[37] Treatment E., Retinopathy D. (1991). Classification of Diabetic Retinopathy from Fluorescein Angiograms: ETDRS Report Number 11. Ophthalmology.

[38] Treatment E., Retinopathy D. (1991). Fundus Photographic Risk Factors for Progression of Diabetic Retinopathy: ETDRS Report Number 12. Ophthalmology.

[39] Treatment E., Retinopathy D. (1991). Grading Diabetic Retinopathy from Stereoscopic Color Fundus Photographs—An Extension of the Modified Airlie House Classification: ETDRS Report Number 10. Ophthalmology.

[40] Bursell S.E., Cavallerano J.D., Cavallerano A.A., Clermont A.C., Birkmire-Peters D., Aiello L.P., Aiello L.M. (2001). Stereo nonmydriatic digital-video color retinal imaging compared with Early Treatment Diabetic Retinopathy Study seven standard field 35-mm stereo color photos for determining level of diabetic retinopathy. Ophthalmology.

[41] Abràmoff M.D., Lou Y., Erginay A., Clarida W., Amelon R., Folk J.C., Niemeijer M. (2016). Improved automated detection of diabetic retinopathy on a publicly available dataset through integration of deep learning. Investig. Ophthalmol. Vis. Sci..

[42] Gulshan V., Peng L., Coram M., Stumpe M.C., Wu D., Narayanaswamy A., Venugopalan S., Widner K., Madams T., Cuadros J. (2016). Development and validation of a deep learning algorithm for detection of diabetic retinopathy in retinal fundus photographs. JAMA—J. Am. Med. Assoc..

[43] Abramoff M.D., Garvin M.K., Sonka M. (2010). Retinal imaging and image analysis. IEEE Rev. Biomed. Eng..

[44] Antonetti D.A., Klein R., Gardner T.W. (2012). Diabetic retinopathy. N. Engl. J. Med..

[45] Simó R., Stitt A.W., Gardner T.W. (2018). Neurodegeneration in diabetic retinopathy: Does it really matter?. Diabetologia.

[46] Jackson G.R., Barber A.J. (2010). Visual dysfunction associated with diabetic retinopathy. Curr. Diab. Rep..

[47] Trento M., Durando O., Lavecchia S., Charrier L., Cavallo F., Costa M.A., Hernández C., Simó R., Porta M. (2017). Vision related quality of life in patients with type 2 diabetes in the EUROCONDOR trial. Endocrine.

[48] Wolff B.E., Bearse M.A., Schneck M.E., Dhamdhere K., Harrison W.W., Barez S., Adams A.J. (2015). Color vision and neuroretinal function in diabetes. Doc. Ophthalmol..

[49] Joltikov K.A., de Castro V.M., Davila J.R., Anand R., Khan S.M., Farbman N., Jackson G.R., Johnson C.A., Gardner T.W. (2017). Multidimensional Functional and Structural Evaluation Reveals Neuroretinal Impairment in Early Diabetic Retinopathy. Invest. Ophthalmol. Vis. Sci..

[50] Reis A., Mateus C., Melo P., Figueira J., Cunha-Vaz J., Castelo-Branco M. (2014). Neuroretinal dysfunction with intact blood-retinal barrier and absent vasculopathy in type 1 diabetes. Diabetes.

[51] Juen S., Kieselbach G.F. (2011). Electrophysiological Changes in Juvenile Diabetics Without Retinopathy. Arch. Ophthalmol..

[52] Di Leo M.A.S., Caputo S., Falsini B., Porciatti V., Greco A.V., Ghirlanda G. (1994). Presence and further development of retinal dysfunction after 3-year follow up in IDDM patients without angiographically documented vasculopathy. Diabetologia.

[53] Tyrberg M., Lindblad U., Melander A., Lövestam-Adrian M., Ponjavic V., Andréasson S. (2011). Electrophysiological studies in newly onset type 2 diabetes without visible vascular retinopathy. Doc. Ophthalmol..

[54] van Dijk H.W., Verbraak F.D., Stehouwer M., Kok P.H.B., Garvin M.K., Sonka M., DeVries J.H., Schlingemann R.O., Abràmoff M.D. (2011). Association of visual function and ganglion cell layer thickness in patients with diabetes mellitus type 1 and no or minimal diabetic retinopathy. Vision Res..

[55] Adams A.J., Bearse M.A. (2012). Retinal neuropathy precedes vasculopathy in diabetes: A function-based opportunity for early treatment intervention?. Clin. Exp. Optom..

[56] Tey K.Y., Teo K., Tan A.C.S., Devarajan K., Tan B., Tan J., Schmetterer L., Ang M. (2019). Optical coherence tomography angiography in diabetic retinopathy: A review of current applications. Eye Vis..

[57] Sohn E.H., Van Dijk H.W., Jiao C., Kok P.H.B.B., Jeong W., Demirkaya N., Garmager A., Wit F., Kucukevcilioglu M., Van Velthoven M.E.J.J. (2016). Retinal neurodegeneration may precede microvascular changes characteristic of diabetic retinopathy in diabetes mellitus. Proc. Natl. Acad. Sci. USA.

[58] Chihara E., Matsuoka T., Ogura Y., Matsumura M. (1993). Retinal nerve fiber layer defect as an early manifestation of diabetic retinopathy. Ophthalmology.

[59] van Dijk H.W., Verbraak F.D., Kok P.H.B., Garvin M.K., Sonka M., Lee K., Devries J.H., Michels R.P.J., van Velthoven M.E.J., Schlingemann R.O. (2010). Decreased retinal ganglion cell layer thickness in patients with type 1 diabetes. Investig. Ophthalmol. Vis. Sci..

[60] Abcouwer S.F., Gardner T.W. (2014). Diabetic retinopathy: Loss of neuroretinal adaptation to the diabetic metabolic environment. Ann. N. Y. Acad. Sci..

[61] Gray E.J., Gardner T.W. (2015). Retinal Failure in Diabetes: A Feature of Retinal Sensory Neuropathy. Curr. Diab. Rep..

[62] Cunha-Vaz J.G. (2015). Diabetic retinopathy: Need for more research to understand the relative role of neuropathy and microvascular disease. Ophthalmic Res..

[63] Antonetti D.A. (2021). The neuroscience of diabetic retinopathy. Vis. Neurosci..

[64] Sun J.K., Aiello L.P., Abràmoff M.D., Antonetti D.A., Dutta S., Pragnell M., Levine S.R., Gardner T.W. (2020). Updating the Staging System for Diabetic Retinal Disease. Ophthalmology.

[65] Bailey R.A., Wang Y., Zhu V., Rupnow M.F. (2014). Chronic kidney disease in US adults with type 2 diabetes: An updated national estimate of prevalence based on Kidney Disease: Improving Global Outcomes (KDIGO) staging. BMC Res. Notes.

[66] Afkarian M., Zelnick L.R., Hall Y.N., Heagerty P.J., Tuttle K., Weiss N.S., de Boer I.H. (2016). Clinical Manifestations of Kidney Disease Among US Adults with Diabetes, 1988-2014. JAMA.

[67] Selby J.V., Friedman G.D., Quesenberry C.P., Weiss N.S. (1992). TA Case–Control Study of Screening Sigmoidoscopy and Mortality from Colorectal Cancer. N. Engl. J. Med..

[68] Viberti G.C., Jarrett R.J., Mahmud U., Hill R.D., Argyropoulos A., Keen H. (1982). Microalbuminuria As a Predictor of Clinical Nephropathy in Insulin-Dependent Diabetes Mellitus. Lancet.

[69] Caramori M.L., Fioretto P., Mauer M. (2000). The need for early predictors of diabetic nephropathy risk: Is albumin excretion rate sufficient?. Diabetes.

[70] MacIsaac R.J., Ekinci E.I. (2019). Progression of diabetic kidney disease in the absence of albuminuria. Diabetes Care.

[71] Lewis E.J., Hunsicker L.G., Clarke W.R., Berl T., Pohl M.A., Lewis J.B., Ritz E., Atkins R.C., Rohde R., Raz I. (2001). Renoprotective effect of the angiotensin-receptor antagonist irbesartan in patients with nephropathy due to type 2 diabetes. N. Engl. J. Med..

[72] Parving H.H., Hommel E., Jensen B.R., Hansen H.P. (2001). Long-term beneficial effect of ACE inhibition on diabetic nephropathy in normotensive type 1 diabetic patients. Kidney Int..

[73] Kasiske B.L., Kalil R.S.N., Ma J.Z., Liao M., Keane W.F. (1993). Effect of antihypertensive therapy on the kidney in patients with diabetes: A meta-regression analysis. Ann. Intern. Med..

[74] Hebert L.A., Bain R.P., Verme D., Cattran D., Whittier F.C., Tolchin N., Rohde R.D., Lewis E.J. (1994). Remission of nephrotic range proteinuria in type I diabetes. Collaborative Study Group. Kidney Int..

[75] Lewis E.J., Hunsicker L.G., Bain R.P., Rohde R.D. (1993). The effect of angiotensin-converting-enzyme inhibition on diabetic nephropathy. The Collaborative Study Group. N. Engl. J. Med..

[76] Neuen B.L., Young T., Heerspink H.J.L., Neal B., Perkovic V., Billot L., Mahaffey K.W., Charytan D.M., Wheeler D.C., Arnott C. (2019). SGLT2 inhibitors for the prevention of kidney failure in patients with type 2 diabetes: A systematic review and meta-analysis. Lancet. Diabetes Endocrinol..

[77] Tuttle K.R., Brosius F.C., Adler S.G., Kretzler M., Mehta R.L., Tumlin J.A., Tanaka Y., Haneda M., Liu J., Silk M.E. (2018). JAK1/JAK2 inhibition by baricitinib in diabetic kidney disease: Results from a Phase 2 randomized controlled clinical trial. Nephrol. Dial. Transplant..

[78] Brosius F.C., Ju W. (2018). The Promise of Systems Biology for Diabetic Kidney Disease. Adv. Chronic Kidney Dis..

[79] Bhensdadia N.M., Hunt K.J., Lopes-Virella M.F., Tucker J.M., Mataria M.R., Alge J.L., Neely B.A., Janech M.G., Arthur J.M. (2013). Veterans Affairs Diabetes Trial (VADT) study group Urine haptoglobin levels predict early renal functional decline in patients with type 2 diabetes. Kidney Int..

[80] Zürbig P., Jerums G., Hovind P., MacIsaac R.J., Mischak H., Nielsen S.E., Panagiotopoulos S., Persson F., Rossing P. (2012). Urinary proteomics for early diagnosis in diabetic nephropathy. Diabetes.

[81] Satirapoj B., Dispan R., Radinahamed P., Kitiyakara C. (2018). Urinary epidermal growth factor, monocyte chemoattractant protein-1 or their ratio as predictors for rapid loss of renal function in type 2 diabetic patients with diabetic kidney disease. BMC Nephrol..

[82] Berthier C.C., Zhang H., Schin M., Henger A., Nelson R.G., Yee B., Boucherot A., Neusser M.A., Cohen C.D., Carter-Su C. (2009). Enhanced expression of janus kinase-signal transducer and activator of transcription pathway members in human diabetic nephropathy. Diabetes.

[83] Woroniecka K.I., Park A.S.D., Mohtat D., Thomas D.B., Pullman J.M., Susztak K. (2011). Transcriptome analysis of human diabetic kidney disease. Diabetes.

[84] Lilly E. (2018). Olumiant (Baricitinib). https://www.accessdata.fda.gov/drugsatfda_docs/label/2018/207924s000lbl.pdf.

[85] Asencio-Duran M., Vallejo-Garcia J.L., Pastora-Salvador N., Fonseca-Sandomingo A., Romano M.R. (2012). Vitreous diagnosis in neoplastic diseases. Mediat. Inflamm..

[86] Ghodasra D.H., Fante R., Gardner T.W., Langue M., Niziol L.M., Besirli C., Cohen S.R., Dedania V.S., Demirci H., Jain N. (2016). Safety and feasibility of quantitative multiplexed cytokine analysis from Office-Based vitreous aspiration. Investig. Ophthalmol. Vis. Sci..

[87] Wong T.Y., Coresh J., Klein R., Muntner P., Couper D.J., Sharrett A.R., Klein B.E.K., Heiss G., Hubbard L.D., Duncan B.B. (2004). Retinal microvascular abnormalities and renal dysfunction: The Atherosclerosis Risk in Communities Study. J. Am. Soc. Nephrol..

[88] Trevisan R., Vedovato M., Mazzon C., Coracina A., Iori E., Tiengo A., Del Prato S. (2002). Concomitance of diabetic retinopathy and proteinuria accelerates the rate of decline of kidney function in type 2 diabetic patients. Diabetes Care.

[89] Manaviat M.R., Afkhami M., Shoja M.R. (2004). Retinopathy and microalbuminuria in type II diabetic patients. BMC Ophthalmol..

[90] Dogné S., Flamion B., Caron N. (2018). Endothelial glycocalyx as a shield against diabetic vascular complications: Involvement of hyaluronan and hyaluronidases. Arterioscler. Thromb. Vasc. Biol..

[91] Leskova W., Pickett H., Eshaq R.S., Shrestha B., Pattillo C.B., Harris N.R. (2019). Effect of diabetes and hyaluronidase on the retinal endothelial glycocalyx in mice. Exp. Eye Res..

[92] Kumase F., Morizane Y., Mohri S., Takasu I., Ohtsuka A., Ohtsukri H. (2010). Glycocalyx degradation in retinal and choroidal capillary endothelium in rats with diabetes and hypertension. Acta Med. Okayama.

[93] To M., Goz A., Camenzind L., Oertle P., Candiello J., Sullivan M., Henrich P.B., Loparic M., Safi F., Eller A. (2013). Diabetes-induced morphological, biomechanical, and compositional changes in ocular basement membranes. Exp. Eye Res..

[94] Zhao L., Zou Y., Liu F. (2020). Transforming Growth Factor-Beta1 in Diabetic Kidney Disease. Front. Cell Dev. Biol..

[95] Ziyadeh F.N., Hoffman B.B., Han D.C., Iglesias-De La M.C.C., Hong S.W., Isono M., Chen S., McGowan T.A., Sharma K. (2000). Long-term prevention of renal insufficiency, excess matrix gene expression, and glomerular mesangial matrix expansion by treatment with monoclonal antitransforming growth factor-β antibody in db/db diabetic mice. Proc. Natl. Acad. Sci. USA.

[96] Loeffler I., Wolf G. (2014). Transforming growth factor-β and the progression of renal disease. Nephrol. Dial. Transplant..

[97] Gerhardinger C., Dagher Z., Sebastiani P., Yong S.P., Lorenzi M. (2009). The transforming growth factor-β pathway is a common target of drugs that prevent experimental diabetic retinopathy. Diabetes.

[98] Pearce I., Simó R., Lövestam-Adrian M., Wong D.T., Evans M. (2019). Association between diabetic eye disease and other complications of diabetes: Implications for care. A systematic review. Diabetes Obes. Metab..

[99] Wilson P.C., Wu H., Kirita Y., Uchimura K., Ledru N., Rennke H.G., Welling P.A., Waikar S.S., Humphreys B.D. (2019). The single-cell transcriptomic landscape of early human diabetic nephropathy. Proc. Natl. Acad. Sci. USA.

[100] Nathan D.M., Genuth S., Lachin J., Cleary P., Crofford O., Davis M., Rand L., Siebert C., Diabetes Control and Complications Trial Research Group (1993). The effect of intensive treatment of diabetes on the development and progression of long-term complications in insulin-dependent diabetes mellitus. N. Engl. J. Med..

[101] Turner R. (1998). Effect of intensive blood-glucose control with metformin on complications in overweight patients with type 2 diabetes (UKPDS 34). Lancet.

[102] Matthews D.R., Stratton I.M., Aldington S.J., Holman R.R., Kohner E.M. (2004). Risks of progression of retinopathy and vision loss related to tight blood pressure control in type 2 diabetes mellitus: UKPDS 69. Arch. Ophthalmol..

[103] Turner R., Holman R., Stratton I., Cull C., Frighi V., Manley S., Matthews D., Neil A., McElroy H., Kohner E. (1998). Tight blood pressure control and risk of macrovascular and microvascular complications in type 2 diabetes: UKPDS 38. UK Prospective Diabetes Study Group. BMJ.

[104] Chew E.Y., Ambrosius W.T., Davis M.D., Danis R.P., Gangaputra S., Greven C.M., Hubbard L., Esser B.A., ACCORD Study Group, ACCORD Eye Study Group (2010). Effects of medical therapies on retinopathy progression in type 2 diabetes. N. Engl. J. Med..

[105] Wilkinson-Berka J.L. (2006). Angiotensin and diabetic retinopathy. Int. J. Biochem. Cell Biol..

[106] Barnett A.H., Bain S.C., Bouter P., Karlberg B., Madsbad S., Jervell J., Mustonen J. (2004). Angiotensin-receptor blockade versus converting-enzyme inhibition in type 2 diabetes and nephropathy. N. Engl. J. Med..

[107] Sharma A., Valle M.L., Beveridge C., Liu Y., Sharma S. (2019). Unraveling the role of genetics in the pathogenesis of diabetic retinopathy. Eye.

[108] Usui-Ouchi A., Friedlander M. (2019). Anti-VEGF therapy: Higher potency and long-lasting antagonism are not necessarily better. J. Clin. Investig..

[109] Maguire M.G., Liu D., Glassman A.R., Jampol L.M., Johnson C.A., Baker C.W., Bressler N.M., Gardner T.W., Pieramici D., Stockdale C.R. (2020). Visual Field Changes over 5 Years in Patients Treated with Panretinal Photocoagulation or Ranibizumab for Proliferative Diabetic Retinopathy. JAMA Ophthalmol..

[110] Nair P., Aiello L.P., Gardner T.W., Jampol L.M., Ferris F.L. (2016). Report from the NEI/FDA diabetic retinopathy clinical trial design and endpoints workshop. Investig. Ophthalmol. Vis. Sci..

[111] Simó R., Hernández C., Porta M., Bandello F., Grauslund J., Harding S.P., Aldington S.J., Egan C., Frydkjaer-Olsen U., García-Arumí J. (2019). Effects of Topically Administered Neuroprotective Drugs in Early Stages of Diabetic Retinopathy: Results of the EUROCONDOR Clinical Trial. Diabetes.

[112] Brigell M.G., Chiang B., Maa A.Y., Davis C.Q. (2020). Enhancing Risk Assessment in Patients with Diabetic Retinopathy by Combining Measures of Retinal Function and Structure. Transl. Vis. Sci. Technol..

[113] Foundation J.D.R. (2019). Complications. http://grantcenter.jdrf.org/wp-content/uploads/2019/07/Complications-Program-Strategy.pdf.

[114] Hodgin J.B., Nair V., Zhang H., Randolph A., Harris R.C., Nelson R.G., Weil E.J., Cavalcoli J.D., Patel J.M., Brosius F.C. (2013). Identification of cross-species shared transcriptional networks of diabetic nephropathy in human and mouse glomeruli. Diabetes.

[115] Fligor C.M., Langer K.B., Sridhar A., Ren Y., Shields P.K., Edler M.C., Ohlemacher S.K., Sluch V.M., Zack D.J., Zhang C. (2018). Three-Dimensional Retinal Organoids Facilitate the Investigation of Retinal Ganglion Cell Development, Organization and Neurite Outgrowth from Human Pluripotent Stem Cells. Sci. Rep..

[116] NIH About Tissue Chip. https://ncats.nih.gov/tissuechip/about.

